# Differential protein expression in chicken macrophages and heterophils in vivo following infection with *Salmonella* Enteritidis

**DOI:** 10.1186/s13567-017-0439-0

**Published:** 2017-06-17

**Authors:** Zuzana Sekelova, Hana Stepanova, Ondrej Polansky, Karolina Varmuzova, Marcela Faldynova, Radek Fedr, Ivan Rychlik, Lenka Vlasatikova

**Affiliations:** 1 Veterinary Research Institute, Hudcova 70, 621 00 Brno, Czech Republic; 2 Department of Cytokinetics, Institute of Biophysics of the CAS, Kralovopolska 135, 612 65 Brno, Czech Republic; 3 Center of Biomolecular and Cellular Engineering, International, Clinical Research Center, St. Anne’s University Hospital Brno, Pekarska 53, 656 91 Brno, Czech Republic

## Abstract

In this study we compared the proteomes of macrophages and heterophils isolated from the spleen 4 days after intravenous infection of chickens with *Salmonella* Enteritidis. Heterophils were characterized by expression of MMP9, MRP126, LECT2, CATHL1, CATHL2, CATHL3, LYG2, LYZ and RSFR. Macrophages specifically expressed receptor proteins, e.g. MRC1L, LRP1, LGALS1, LRPAP1 and a DMBT1L. Following infection, heterophils decreased ALB and FN1, and released MMP9 to enable their translocation to the site of infection. In addition, the endoplasmic reticulum proteins increased in heterophils which resulted in the release of granular proteins. Since transcription of genes encoding granular proteins did not decrease, these genes remained continuously transcribed and translated even after initial degranulation. Macrophages increased amounts of fatty acid elongation pathway proteins, lysosomal and phagosomal proteins. Macrophages were less responsive to acute infection than heterophils and an increase in proteins like CATHL1, CATHL2, RSFR, LECT2 and GAL1 in the absence of any change in their expression at RNA level could even be explained by capturing these proteins from the external environment into which these could have been released by heterophils.

**Electronic supplementary material:**

The online version of this article (doi:10.1186/s13567-017-0439-0) contains supplementary material, which is available to authorized users.

## Introduction

Macrophages and heterophils represent professional phagocytes acting as effectors and modulators of innate immunity as well as orchestrators of adaptive immunity [1]. Heterophils, the avian counterparts of mammalian neutrophils, belong among the first responders to bacterial infections and sensing of pathogen associated molecular patterns (PAMPs) stimulates heterophils for phagocytosis as well as release of bactericidal proteins stored in heterophil granules into the extracellular environment [2]. In agreement with their general function in host protection against pathogens, heterophils play a crucial role in the protection of chickens against *Salmonella* infection and chickens with heterophil depletion are not protected against colonization of systemic sites [3–5]. However, although there are several reports on specific heterophil functions during infection of chickens with *Salmonella enterica*, their genome-wide response to infection has not been characterized so far.

Macrophages are professional phagocytes responsible for the destruction and clearance of pathogens. When activated, macrophages increase their antibacterial activity by the expression of antimicrobial peptides like cathepsins B, C, D and S, avidin, ferritin or ovotransferrin [6], and production of NO radicals from arginine by inducible NO synthase. The antimicrobial proteins expressed by macrophages are commonly produced also by heterophils though it is not known to what extent these may differ in their immediate availability and total amount produced by both cell types. Macrophages can also regulate the immune response by the expression of cytokines e.g. IL1β, IL6, IL8, IL18 or LITAF [7] and are capable of antigen presentation [8–10]. However, similar to heterophils, an unbiased report on total proteome expressed by chicken macrophages is absent.

In our previous study we showed that heterophils and macrophages increase in the spleen of chickens when intravenously infected with *Salmonella* Enteritidis (*S.* Enteritidis) [7]. Next we characterized the gene expression at the tissue level in the whole spleen and expression of selected transcripts was tested in sorted leukocyte subpopulations [6]. However, none of this provided general data on the protein expression in chicken heterophils and macrophages. Although intravenous infection of chickens only partially represents specific *Salmonella*—chicken interactions which are mixed up with a general response to bacteremia caused by Gram negative bacterium, this way of infection represents a model for the understanding heterophil and macrophage functions during early response to infection. In the current study we therefore isolated heterophils and macrophages from chicken spleens by fluorescence-activated cell sorting (FACS), purified proteins from these cells and identified them by mass spectrometry. This allowed us to (1) characterize the total proteome of heterophils and macrophages, (2) define proteins which exhibited differential abundance in chicken heterophils compared to macrophages and (3) identify proteins that changed in abundance following the intravenous infection with *S.* Enteritidis in either of these populations. Since we also included a group of chickens which was vaccinated prior to challenge, we also addressed whether there are any proteins specifically expressed by the macrophages or heterophils from the vaccinated chickens. Using this approach we identified over one hundred proteins characteristic of either chicken heterophils or macrophages which allowed us to further refine their function in chickens.

## Materials and methods

### Ethics statement

The handling of animals in this study was performed in accordance with current Czech legislation (Animal protection and welfare Act No. 246/1992 Coll. of the Government of the Czech Republic). The specific experiments were approved by the Ethics Committee of the Veterinary Research Institute (permit number 5/2013) followed by the Committee for Animal Welfare of the Ministry of Agriculture of the Czech Republic (permit number MZe 1480).

### Bacterial strains and chicken line

Newly hatched ISA Brown chickens from an egg laying line (Hendrix Genetics, Netherlands) were used in this study. Chickens were reared in perforated plastic boxes with free access to water and feed and each experimental or control group was kept in a separate room. The chickens were vaccinated with *S.* Enteritidis mutant completely lacking *Salmonella* pathogenicity island 1 (SPI-1) constructed as described earlier [11] and infected with isogenic wild type *S.* Enteritidis 147 spontaneously resistant to nalidixic acid. The strains were grown in LB broth at 37 °C for 18 h followed by pelleting bacteria at 10 000 × *g* for 1 min and re-suspending the pellet in the same volume of PBS as was the original volume of LB broth.

### Experimental infection

There were 3 groups of chickens. Six chickens from the control group were sacrificed on day 48 of life. An additional 6 chickens (group 2) were infected intravenously with 10^7^ CFU of wild type *S.* Enteritidis in 0.1 mL PBS on day 44 of life. The last 6 chickens (group 3) were orally vaccinated on day 1, revaccinated on day 21 of life with 10^7^ CFU of *S.* Enteritidis SPI-1 mutant in 0.1 mL of inoculum and challenged intravenously with 10^7^ CFU of wild type *S.* Enteritidis on day 44 of life. Intravenous mode of infection was used mainly to stimulate macrophage and heterophil response rather than to model natural infection of chickens with *S.* Enteritidis. All chickens in groups 2 and 3 were sacrificed 4 days post infection, i.e. when aged 48 days. The spleens from the chickens from all three groups were collected into PBS during necropsy. To confirm *S.* Enteritidis infection, approximately 0.5 g of liver tissue was homogenised in 5 mL of peptone water, tenfold serially diluted and plated in XLD agar, as described previously [11].

### Collecting heterophil and macrophage subpopulations by flow cytometry

The cell suspensions were prepared by pressing the spleen tissue through a fine nylon mesh followed by 2 washes with 30 mL of cold PBS. After the last washing step, the splenic leukocytes were re-suspended in 1 mL of PBS and used for surface marker staining.

In total 10^8^ of cells were incubated for 20 min with anti-monocyte/macrophage:FITC (clone KUL01 from Southern Biotech) and CD45:APC (clone LT40 from Southern Biotech), followed by wash with PBS. Monocytes/macrophages (CD45+KUL01+) and heterophils (identified based on FSC/SSC characteristics within CD45+ cells) were sorted using a FACSFusion flow cytometer operated by FACSDiva software (BD Biosciences). Only for simplicity, the monocytes/macrophages population will be called as “macrophage (Ma)” in the rest of this paper. Sorted cells were collected in PBS and immediately processed as described below. A small aliquot from each sample was subjected to immediate purity analysis. The purity of macrophages was 88.6 ± 5.3% and of heterophils 88.1 ± 4.2% when counting cell of expected staining, and FSC and SSC parameters out of all particles. When we gated at the area with live cells, the purity of macrophages and heterophils was between 97 and 98%. Majority of contaminants therefore represented cellular debris and only around 2.5% of contaminants were formed by non-target cells.

### Protein and RNA isolation from sorted cells, reverse transcription of mRNA and quantitative real time PCR (qPCR)

Sorted leukocyte subpopulations were lysed in 500 µL of Tri Reagent (MRC) for parallel isolation of RNA and proteins. Upon addition of 4-bromoanisole and 15 min centrifugation at 14 000 × *g*, proteins were precipitated with acetone from the lower organic phase. RNA present in upper aqueous phase was further purified using RNeasy purification columns according to the instructions of the manufacturer (Qiagen). The concentration of RNA was determined spectrophotometrically (Nanodrop, Thermo Scientific) and 1 µg of RNA was immediately reverse transcribed into cDNA using MuMLV reverse transcriptase (Invitrogen) and oligo dT primers. After reverse transcription, the cDNA was diluted 10 times with sterile water and stored at −20 °C prior qPCR. qPCR was performed in 3 µL volumes in 384-well microplates using QuantiTect SYBR Green PCR Master Mix (Qiagen) and a Nanodrop pipetting station from Innovadyne for PCR mix dispensing following MIQE recommendations [12]. Amplification of PCR products and signal detection were performed using a LightCycler II (Roche) with an initial denaturation at 95 °C for 15 min followed by 40 cycles of 95 °C for 20 s, 60 °C for 30 s and 72 °C for 30 s, followed by the determination of melting temperature of resulting PCR products to exclude false positive amplification. Each sample was subjected to qPCR in duplicate and the mean values of the Cq values of genes of interest were normalized (ΔCt) to an average Cq value of three reference genes (GAPDH, TBP and UB). The relative expression of each gene of interest was finally calculated as 2^−ΔCq^. Statistical analysis using a two sample t test for means equality was performed when comparing levels of mRNA expression between chicken groups and results with *p* value ≤ 0.05 were considered as significantly different in expression. Sequence of reference genes GAPDH, TBP and UB have been published elsewhere [13, 14]. Sequences of all newly designed primers used in this study including their location within different exons and sizes of PCR products are listed in Additional file 1.

### Sample preparation for LC–MS/MS analysis

Precipitated proteins were washed with acetone and dried. The pellets were dissolved in 300 µL of 8 M urea and processed by the filter aided sample preparation method [15] using Vivacon 10 kDa MWCO filter (Sartorius Stedim Biotech). Proteins were washed twice with 100 µL of 8 M urea and reduced by 100 µL of 10 mM DTT. After reduction, proteins were incubated with 100 µL of 50 mM IAA and washed twice with 100 µL of 25 mM TEAB. Trypsin (Promega) was used at 1:50 ratio (w/w) and the digestion proceeded for 16 h at 30 °C.

For comparative analysis, peptide concentration was determined spectrophotometrically (Nanodrop, Thermo Scientific) and samples from the same group of chickens were pooled. Pooled samples were then labelled using the stable isotope dimethyl labelling protocol as described previously [16]. Labeled samples were mixed and 3 subfractions were prepared using Oasis MCX Extraction Cartridges (Waters). The samples were desalted on SPE C18 Extraction Cartridges (Empore) and concentrated in a SpeedVac (Thermo Scientific) prior to LC–MS/MS.

### LC–MS/MS analysis

Protein samples were analysed on LC–MS/MS system using an UltiMate 3000 RSLCnano liquid chromatograph (Dionex) connected to LTQ-Orbitrap Velos Pro mass spectrometer (Thermo Scientific). Chromatographic separation was performed on EASY-Spray C18 separation column (25 cm × 75 µm, 3 µm particles, Thermo Scientific) with 2 h long (label free) or 3 h long (label based) 3–36% acetonitrile gradient.

High resolution (30 000 FWHM at 400 *m*/*z*) MS spectra were acquired for the 390–1700 *m*/*z* interval in an Orbitrap analyser with an AGC target value of 1 × 10^6^ ions and maximal injection time of 100 ms. Low resolution MS/MS spectra were acquired in Linear Ion Trap in a data-dependent manner and the top 10 precursors exceeding a threshold of 10 000 counts and having a charge state of +2 or +3 were isolated within a 2 Da window and fragmented using CID.

### Data processing, protein identification and quantification

Raw data were analysed using the Proteome Discoverer (v.1.4). MS/MS spectra identification was performed by SEQUEST using the *Gallus gallus* protein sequences obtained from Uniprot database. Precursor and fragment mass tolerance were 10 ppm and 0.6 Da, respectively. Carbamidomethylation (C) and oxidation (M) were set as static and dynamic modifications, respectively. Dimethylation (N-term and K) was set as static modification in the label-based analysis. Only peptides with a false discovery rate FDR ≤ 5% were used for protein identification.

Spectral counting, the protocol in which abundance of a protein is expressed as the total number of tandem mass spectra matching its peptides (peptide spectrum matches, PSM), was used for comparative label-free analysis of heterophil and macrophage proteomes [17]. For a general comparison of protein abundance between heterophils and macrophages, PSMs belonging to a particular protein from all three groups of chickens, i.e. 18 samples, were summed up. The identification of at least two distinct peptides belonging to the particular protein and the threshold of at least 5 PSMs in at least one sample was required for its reliable identification [18, 19]. All data were normalized to the total number of PSMs in individual samples. Statistical analysis using a *t* test was performed and the proteins with *p* value ≤ 0.05 and with at least four fold differences in its amounts were considered as significantly different in their abundance between the subpopulations.

In the label-based quantification, only unique peptide sequences with at least 20 PSMs were considered for peptide ratio calculations. Subsequent analysis of label-based data was performed in R (https://www.R-project.org). For each protein, its individual peptide ratios were log_2_ transformed, mean values were calculated and tested with a one sample *t* test. Benjamini-Hochberg correction for multiple testing was then applied to the obtained *p* values. Only proteins having ≥ twofold change and adjusted *p* value ≤ 0.05 were considered as being significantly different in abundance.

### Bioinformatic analysis

Protein interaction networks were built using the online database resource Search Tool for the Retrieval of Interacting Genes (STRING). Proteins were further analyzed using Gene Ontology (GO) database and the Kyoto Encyclopedia of Genes and Genomes (KEGG) for their classification into specific pathways. PCA plots were calculated and created in R (https://www.R-project.org).

## Results

### *S.* Enteritidis infection

Intravenous *S.* Enteritidis infection resulted in a high colonization of systemic sites. Average log_10_ *S.* Enteritidis counts were 5.03 ± 0.54 and 3.06 ± 0.99 CFU/g of liver in the infected chickens and the vaccinated and infected chickens, respectively. Despite this, no fatalities were observed among infected chickens. No *S.* Enteritidis was detected in any of the control non-infected chickens.

### Identification of heterophil and macrophage specific proteins

Proteins specific for chicken heterophils or macrophages were determined irrespective whether these were obtained from the infected or non-infected chickens.

Altogether, 858 proteins from heterophils and 1032 proteins from macrophages were detected. Out of these, 654 proteins were expressed both in heterophils and macrophages. Two-hundred and eight proteins were detected in macrophages only and an additional 126 proteins were 4 times or more abundant in macrophages than in heterophils. On the other hand, 34 proteins were detected in heterophils only and an additional 44 proteins were 4 times or more abundant in heterophils than in macrophages (Additional file 2).

### Proteins characteristic for heterophils

Out of 78 proteins characteristic for heterophils (Additional file 2), 20 with the highest PSM difference between heterophils and macrophages are listed in Table 1. These included MRP126, LECT2, CATHL1, CATHL2, CATHL3, LYG2, LYZ and RSFR proteins, all with antibacterial functions. STOM and RAB27A proteins controlling storage and release of granular proteins in neutrophils also belonged among the characteristic and highly expressed proteins in heterophils. Two serine protease inhibitors, SERPINB10 and SERPINB1, were also found among the 20 most characteristic heterophil proteins (Table 1). Only a single KEGG pathway was specifically enriched in heterophils and this was the starch and sucrose metabolism pathway comprising PYGL, PGM1 and PGM2 proteins (*p* = 1.7E−4). Despite the KEGG pathway designation, all these proteins represent enzymes involved in glycogen metabolism [20].

**Table 1:** **Twenty most characteristic proteins of heterophils (Het) compared to macrophages (Ma)**

Acc. no.	Protein name	Gene ID	∆PSM^a^	Fold ratio Het:Ma	Response to the infection	Function
P28318	MRP126, calprotectin	MRP126	7170	9.07	No	Calcium and zinc binding
P08940	Myeloid protein 1	LECT2	5532	6.32	Decrease	Chemotactic factor for Het
P02789	Ovotransferrin	OTFB	2351	4.87	Decrease	Iron binding, immune response
O73790	Heterochromatin-associated protein MENT	SERPINB10	1760	6.00	No	DNA condensation, cysteine protease inhibitor
E1C0K1	Extracellular fatty acid-binding protein	ExFABP	1742	4.94	No	Fatty acid and bacterial siderophores binding
F1NG13	Transglutaminase 3	TGM3	1572	19.94	No	Transglutaminase
Q2IAL7	Cathelicidin 2	CATHL2	1402	7.49	Decrease	Antimicrobial peptide
P27042	Lysozyme G	LYG2	989	4.57	Decrease	Antimicrobial peptide
Q2IAL6	Cathelicidin 3	CATHL3	936	5.37	No	Antimicrobial peptide
P00698	Lysozyme C	LYZ	839	5.17	Decrease	Antimicrobial peptide
Q6QLQ5	Cathelicidin 1	CATHL1	833	4.62	Decrease	Antimicrobial peptide
E1BTH1	Leukocyte elastase inhibitor	SERPINB1	627	Only Het	Decrease	Protection against own proteases
F1P284	Leukotriene A(4) hydrolase	LTA4H	603	5.78	Decrease	Epoxide hydrolase and aminopeptidase
F1NGT3	Matrix metallopeptidase 9	MMP9	600	Only Het	Decrease	Degradation of the extracellular matrix
F2Z4L6	Serum albumin	ALB	557	4.79	Decrease	Plasma carrier
P30374	Ribonuclease homolog	RSFR	548	6.89	Decrease	Lysosomal cysteine protease
R9PXN7	Hematopoietic prostaglandin D synthase	HPGDS	504	17.79	No	Cytosolic glutathione S-transferases
E1BTV1	Stomatin	STOM	502	23.82	No	Integral membrane protein
D2D3P4	Rab27a	Rab27a	435	88.08	No	Small GTPase, exocytosis
R4GI24	Integrin alpha-D	ITGAD	379	7.73	No	Adhesion of leukocytes

^a^The difference in PSM counts of particular protein in Het and Ma.

### Proteins characteristic for macrophages

Out of 334 proteins specific for macrophages (Additional file 2), 20 with the highest PSM difference between macrophages and heterophils are listed in Table 2. Five of these represented receptor proteins MRC1L, LRP1, LGALS1, LRPAP1 and DMBT1L, the last one containing the scavenger receptor cysteine-rich (SRCR) domain. CTSB, CKB, MECR, PHB2, H9KZK0 and p41/Li are involved in phagocytosis and antigen presentation. An additional 4 proteins UQCR, UQCRC1, ACO2 and HADHB are localized to the mitochondria. Only 3 proteins, MRC1L, HSP70 and p41/Li, were already recorded in chicken macrophages [21–23] although except for NAT3, PLB and SSB, the expression of the remaining proteins (out of the most abundant listed in Table 2) has been already recorded in murine or human macrophages. Proteins enriched in macrophages belonged to oxidative phosphorylation (*p* = 4.7E−8), fatty acid metabolism (*p* = 1.73E−6), citrate cycle (*p* = 4.2E−6), arginine and proline metabolism (*p* = 8.5E−8) and proteasome (*p* = 4.5E−4).

**Table 2:** **Twenty most characteristic proteins for macrophages (Ma) compared to heterophils (Het)**

Acc. no.	Protein name	Gene ID	∆PSM^a^	Fold ratio Ma:Het	Response to the infection	Function
M1XGZ4	Macrophage mannose receptor 1 like	MRC1L	993	Only Ma	No	C-Type lectin
P98157	Low-density lipoprotein receptor-related protein 1	LRP1	810	Only Ma	No	Endocytic receptor
P07583	Galectin 1	LGALS1	607	Only Ma	No	Beta-galactoside-binding lectin
P43233	Cathepsin B	CTSB	538	8.42	Increase	Cysteine protease
F1NZ86	Heat shock 70 protein, mortalin	HSP70	508	5.30	No	Chaperon
P05122	Creatine kinase B-type	CKB	467	34.77	No	Energy transduction
F1NDD6	LDL receptor related protein associated protein 1	LRPAP1	374	Only Ma	No	LDL receptors trafficking
F1NIX4	Trans-2-enoyl-CoA reductase	MECR	356	33.16	Increase	Fatty acid elongation
F1P180	Aspartate aminotransferase	GOT2	350	7.27	No	Transaminase
P13914	Arylamine *N*-acetyltransferase	NAT3	350	23.92	No	Conjugating enzyme
H9KZK0	Protein containing the scavenger receptor cysteine-rich (SRCR) domain	DMBT1L	318	Only Ma	No	Scavenger receptor
E1BZF7	Putative phospholipase B	PLB	317	6.23	No	Removing fatty acids from phospholipids
Q6J613	Invariant chain isoform p41	Li	312	6.87	No	Chaperone
F1P582	Mitochondrial ubiquinol-cytochrome-c reductase complex core protein 2	UQCR	309	4.36	No	Oxidative phosphorylation
Q5ZMW1	Aconitate hydratase, mitochondrial	ACO2	306	6.17	No	TCA cycle
F1NAC6	Cytochrome b-c1 complex subunit 1	UQCRC1	289	6.42	No	Oxidative phosphorylation
F6R1X6	Lupus la protein	SSB	288	6.90	No	Protecting of 3′ poly(U) terminus of transcribed RNA
E1BTT4	Trifunctional enzyme subunit beta, mitochondrial	HADHB	287	30.61	Increase	β-Oxidation of fatty acids
Q5ZMN3	Prohibitin-2	PHB2	282	10.52	No	Not clear
F1NJD6	Guanine deaminase, cypin	GDA	275	Only Ma	No	Oxidizes hypoxanthine to xanthine

^a^The difference in PSM counts of particular protein in Ma and Het.

### Heterophil proteins responding to in vivo infection with *S.* Enteritidis

Altogether, 153 proteins were present in different abundance in the heterophils before and after *S.* Enteritidis infection. Of these, 109 proteins increased and 44 proteins decreased in abundance (Additional files 3 and 4 for all quantified heterophil proteins). Proteins belonging to 2 KEGG categories were enriched in heterophils following *S.* Enteritidis infection. These included the category translation with 39 proteins (*p* = 2.58E−62) and protein processing in endoplasmic reticulum (12 proteins, *p* = 1.74E−11). Twenty proteins with the highest increase in abundance, except for those belonging to the category translation, are listed in Table 3. Among others, these included AVD, F13A, ANXA2, ANXA7 or CTSC.

**Table 3:** **Proteins which increased in abundance in heterophils in response to** ***S.*** **Enteritidis infection**

Acc. no.	Protein name	Gene ID	Fold ratio Inf: noninf	Fold ratio vac: noninf	Function
P02701	Avidin	AVD	55.57*	32.06*	Biotin binding
F1P4F4	Translocon-associated protein	SSR1	9.22*	6.36	Protein translocase
P17785	Annexin A2	ANXA2	6.44*	2.11	Activates macrophages for cytokine production
E1BWG1	Coagulation factor XIIIA	F13A	5.63*	2.60*	Crosslinking of fibrin chains, entrapment of bacteria
R4GJX3	Interferon-induced transmembrane protein	IFITM	4.99*	1.73	Acidification of the endosomal compartments, mediator of the host antiviral response
F1NK96	Protein disulfide-isomerase A6	PDIA6	4.33*	2.66*	Protein foldase
F1NVA4	Nucleophosmin	NPM1	3.68*	1.87	Alarmin, nuclear chaperon
F1NT28	Inorganic pyrophosphatase	PPA1	3.52*	1.67	Hydrolysis of inorganic pyrophosphate (PPi)
Q90593	78 kDa glucose-regulated protein	BiP	3.44*	1.94	Chaperon
F1NWB7	Endoplasmin	HSP90B1	3.33*	1.99	Chaperon
E1C1D1	Annexin 7	ANXA7	3.27*	2.68*	Granular membranes fusion and degranulation
P24367	Peptidyl-prolyl cis–trans isomerase B	PPIB	3.26*	2.23*	Regulation of protein folding and maturation
E1C2S1	Talin-1	TLN1	3.12*	2.56*	Activation of neutrophils
Q49B65	EF hand-containing protein 1	EFHD1	3.12*	1.72	Calcium binding
F1NWG2	Cathepsin C	CTSC	3.10*	1.99	Activates serine proteases (elastase, cathepsin G and granzymes)
F1NDY9	Protein disulfide-isomerase A4	PDIA4	2.93*	1.86	Protein foldase
E1C8M9	Calnexin	CANX	2.88*	1.75	Integral protein of the endoplasmic reticulum
E1BQN9	Calcyclin-binding protein	CACYBP	2.88*	2.38*	Calcium-dependent ubiquitination
H9L340	ATP synthase subunit beta	ATP5B	2.82*	1.56	Energy metabolism
F1NB92	Endoplasmic reticulum aminopeptidase 1	ERAP1	2.78*	0.89	Antigen processing and presentation of endogenous peptide via MHC class I

* Significantly different from the expression in heterophils from the non-infected chickens.

Forty-four proteins decreased in abundance in heterophils following *S.* Enteritidis infection and 20 of these with the highest decrease are listed in Table 4. Proteins with decreased abundance were those found in heterophil granules such as MPO, LYZ, LYG2, CTSG, CTSL1, CATHL1, CATHL2, RSFR, MMP9 and LECT2. Another set of proteins which decreased in heterophils following *S.* Enteritidis infection included ALB, FN1 and OTFB (Table 4).

**Table 4:** **List of proteins which decreased in abundance in heterophils in response to** ***S.*** **Enteritidis infection**

Acc. no.	Protein name	Gene ID	Fold ratio inf: noninf	Fold ratio vac: noninf	Function
F1P1U6	Myeloperoxidase	MPO	0.013*	0.071*	Oxidative burst
E1C677	Natural killer cell activator	Gga.18306	0.026*	0.21*	GO prediction: regulation of cytokine biosynthetic process
F1NJT3	Fibronectin	FN1	0.11*	0.56	Binds components of extracellular matrix
F1NFQ7	Serine protease 57	PRSSL1	0.15*	0.37*	Serine-type endopeptidase activity
P00698	Lysozyme C	LYZ	0.16*	0.37*	Antimicrobial peptide
H9L027	Cathepsin G	CTSG	0.19*	0.30*	Lysosomal cysteine protease
Q6QLQ5	Cathelicidin-1	CATHL1	0.20*	0.51	Bactericidal, fungicidal and immunomodulatory activity
F1NZ37	Cathepsin L1	CTSL1	0.22*	0.48*	Controlling element of neutrophil elastase activity
P30374	Ribonuclease homolog	RSFR	0.23*	0.51	Lysosomal cysteine protease
P27042	Lysozyme G	LYG2	0.24*	0.60	Antimicrobial peptide
F2Z4L6	Serum albumin	ALB	0.24*	0.67	Plasma carrier
P02789	Ovotransferrin	OTFB	0.26*	0.55	Iron binding, immune response
F1NGT3	Matrix metallopeptidase 9	MMP9	0.26*	0.77	Degradation of the extracellular matrix
F1NVM1	G-protein coupled receptor 97	GPR97	0.27*	0.66	Regulates migration
Q2IAL7	Cathelicidin-2	CATHL2	0.31*	0.78	Antimicrobial peptide
Q2UZR2	Phosphoglucomutase 1	PGM1	0.35*	0.43*	Glucose metabolic process
E1BZS2	Nucleosome assembly protein 1-like	NAP1L1	0.36*	0.22*	Chaperone for the linker histone
P08940	Myeloid protein 1	LECT2	0.37*	0.62	Chemotactic factor
R4GH86	Glutathione peroxidase	GPX	0.41*	0.57	Protects organism from oxidative damage
F1NYH8	Ena/VASP-like protein	EVL	0.42*	0.70	Regulators of the actin cytoskeleton and cell migration

* Significantly different from the expression in heterophils from the non-infected chickens.

### Macrophage proteins responding to in vivo infection with *S.* Enteritidis

Four KEGG pathways were specifically enriched when testing proteins of increased abundance in macrophages following *S.* Enteritidis infection. These included fatty acid elongation pathway (MECR and HADHB proteins, *p* = 2.49E−4), lysosomal proteins CTSB and CTSC (*p* = 6.98E−3), phagosomal proteins RAB7A and STX7 (*p* = 9.23E−3) and LDHA and HADHB from the microbial metabolism in diverse environments pathway (*p* = 9.4E−3). Other proteins with increased abundance in macrophages following *S.* Enteritidis infection were MRP126, CATHL1, CATHL2, GAL1, CTSB, CTSC, RSFR, SOD1, LECT2, LY86 and FTH, all with antibacterial functions (Table 5). Proteins which decreased in abundance in macrophages following *S.* Enteritidis infection included RBMX, NDUFA4, FNBP1, FAM107, STMN1, GLOD4 and OLA1 (Table 5; Additional files 5, 6 for all quantified macrophage proteins).

**Table 5:** **Proteins of increased or decreased abundance in macrophages in response to** ***S.*** **Enteritidis infection**

Acc. no.	Protein name	Gene ID	Fold ratio inf:noninf	Fold ratio vac:noninf	Function
P28318	MRP126, calprotectin	MRP126	15.67*	5.01*	Calcium and zinc binding
Q6QLQ5	Cathelicidin-1	CATHL1	7.32*	2.95*	Antimicrobial peptide
P30374	Ribonuclease homolog	RSFR	5.84*	1.66	Lysosomal cysteine protease
F1NIX4	Trans-2-enoyl-CoA reductase	MECR	5.47*	3.99*	Fatty acid elongation
P46156	Gallinacin 1	GAL1	4.15*	1.12	Antimicrobial protein
F1N8Q1	Superoxide dismutase	SOD1	4.01*	2.58	Oxygen scavenger
P08940	Myeloid protein 1	LECT2	3.87*	1.35	Chemotactic factor for Het
F1P4F3	Lymphocyte antigen 86, MD-1	LY86	3.53*	3.03	Inhibits LPS response of immune cells
F1NS91	60S ribosomal protein L9	RPL9	3.51*	3.82	Structural part of ribosome
E1BTT4	Trifunctional enzyme subunit beta, mitochondrial	HADHB	3.38*	3.54*	β-Oxidation of fatty acids
P43233	Cathepsin B	CTSB	2.88*	2.57*	Lysosomal cysteine protease
B4X9P4	Microsomal glutathione S-transferase 1	MGST1	2.87*	1.46	Membrane protection from oxidative stress
Q5ZMP2	Syntaxin 7	STX7	2.72*	2.94*	Late endosome–lysosome fusion
E1C0F3	Ras-related protein Rab-7a	RAB7A	2.69*	2.38*	Involved in endocytosis, phagosome–lysosome fusion
F1N9J7	Tubulin alpha-3 chain	Tuba3a	2.63*	1.96	Major constituent of microtubules
P08267	Ferritin heavy chain	FTH	2.62*	2.33*	Storage of iron in a soluble, nontoxic state
P02263	Histone H2A-IV	H2A4	2.61*	3.64*	Formation of nucleosome
F1NWG2	Cathepsin C	CTSC	2.48*	2.46*	Activates serine proteases
Q2IAL7	Cathelicidin-2	CATHL2	2.45*	1.01	Antimicrobial peptide
Q6EE32	Calreticulin	CALR	2.33*	2.21*	Molecular chaperon
Q9I9D1	Voltage-dependent anion-selective channel protein 2	VDAC2	2.27*	2.07*	Inhibits mitochondrial way of apoptosis
P02607	Myosin light polypeptide 6	MYL6	2.7*	1.66	Found in phagosome
F1NB92	Endoplasmic reticulum aminopeptidase 1	ERAP1	2.21*	2.04	Antigen processing and presentation of endogenous peptide via MHC class I
E1BTT8	Lactate dehydrogenase A	LDHA	2.07*	1.71	Glycolysis
R4GM10	Fructose-bisphosphate aldolase C	ALDOC	2.07*	2.33	Glycolysis
P24367	Peptidyl-prolyl cis–trans isomerase B	PPIB	2.00*	0.97	Regulation of protein folding and maturation
Q5ZKQ9	RNA binding motif protein, X-linked	RBMX	0.49*	0.59	Regulation of pre- and post-transcriptional processes
R4GGZ2	NADH dehydrogenase [ubiquinone] 1 alpha subcomplex subunit 4	NDUFA4	0.38*	0.65	Oxidative phosphorylation
E1BYF8	Formin-binding protein 1	FNBP1	0.33*	0.47*	Role in late stage of clathrin-mediated endocytosis
R4GJP1	Family with sequence similarity 107, member B	FAM107	0.32*	0.30*	Candidate tumor suppressor gene
P31395	Stathmin 1	STMN1	0.27*	0.51	Promotes disassembly of microtubules
E1BQI4	Glyoxalase domain-containing protein 4	GLOD4	0.21*	0.18*	Unknown
Q5ZM25	Obg-like ATPase 1	OLA1	0.12*	0.11	Negative role in cell adhesion and spreading

* Significantly different from the expression in macrophages from the non-infected chickens.

### RNA expression

Finally we verified the expression of 37 genes coding for selected proteins listed in Tables 1, 2, 3, 4 and 5. Expression of 4 genes, LRP1, MPO, PPIB and TUBA3A was too low and these genes were excluded from further consideration (Additional file 7).

Six genes (LGALS1, MRC1L, GDA, MECR, DMBT1, LRPAP1) out of 7 proteins selected as specific for macrophages were transcribed in macrophages at a higher level than in heterophils. Only HSP70 was transcribed in macrophages and heterophils at the same level though it was present in higher abundance at the protein level in macrophages. Nine genes (MRP126, OTFB, LYG2, LYZ, SERPINB1, CATHL1, CATHL2, MMP9, LECT2) out of 14 heterophil specific proteins were transcribed in heterophils at a higher level than in macrophages. Two genes of this group (GPX, CTSG) were transcribed in heterophils and macrophages at the same level and the remaining 2 genes (RSFR, LTA4H) were transcribed at a higher level in macrophages though protein mass spectrometry indicated their higher abundance in heterophils.

Expression of 11 proteins which increased in abundance in macrophages following infection of chickens with *S.* Enteritidis was also tested at the RNA level. Except for MRP126, 10 of these (MECR, CTSC, ERAP1, RSFR, SOD1, CALR, CATHL1, CATHL2, LECT2, GAL1) did not exhibit any difference at the transcriptional level. 6 of 7 proteins (ANXA2, F13A, CTSC, ERAP1, AVD, HSP90B1) exhibiting an increased abundance in heterophils following infection of chickens with *S.* Enteritidis, also increased their expression at the level of transcription. Only IFITM did not change its expression at the RNA level. Finally we verified the expression of 11 proteins which decreased in abundance in heterophils following infection of chickens with *S.* Enteritidis. Eight of them (FN1, ALB, CTSL1, OTFB, LYZ, CATHL1, MMP9, LECT2) did not change their expression at the level of transcription and transcripts of 3 of them (RSFR, LYG2, CSTC) even increased following infection.

Similar to the results of protein mass spectrometry, RNA levels of the tested genes in the heterophils or macrophages from the vaccinated chickens were in between the expression in non-infected chickens and chickens infected without previous vaccination. Only 3 genes in heterophils did not follow this scheme and CATHL1, CATHL2 and LECT2 were expressed in heterophils from the vaccinated chickens at significantly higher level than in the heterophils from infected chickens.

## Discussion

Until now, chicken heterophils and macrophages have been characterized only by their specific characteristics like cytokine signaling or production of antimicrobial peptides [2, 6, 7, 24, 25] and an unbiased report characterizing their total proteome, before and after infection, has been missing. In the current study we therefore isolated proteins from heterophils and macrophages and quantified their abundance before and after infection with *S.* Enteritidis by mass spectrometry. We have to remind that mass spectrometry provides reliable data for approximately 800 the most abundant proteins. The lowly represented proteins, despite their potential specificity or responsiveness to infection, could not be therefore detected.

**Figure 1:**
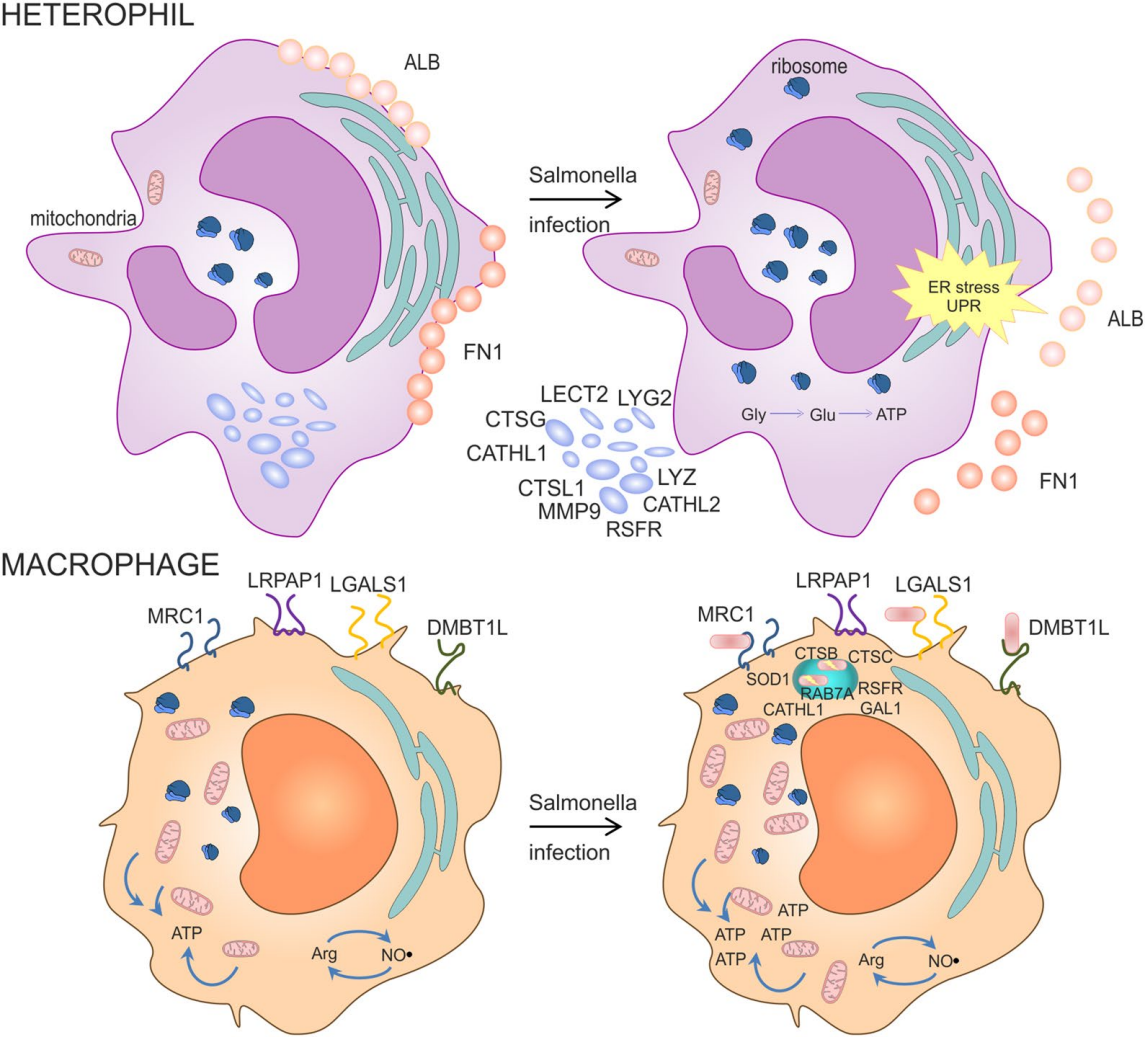
**The most characteristic proteins and their functions in chicken heterophils and macrophages.** Heterophils express MMP9, MRP126, LECT2, CATHL1, CATHL2, CATHL3, LYG2, LYZ and RSFR proteins. Following *S.* Enteritidis infection, heterophils decreased fibrinogen FN1 and albumin ALB, and increased ribosomal proteins. In addition, endoplasmic reticulum proteins are activated which results in the release of granular proteins. Heterophils expressed glycogen (Gly) metabolism pathway which allows for rapid glucose (Glu) availability and anaerobic ATP generation via glycolysis while macrophages increased mitochondrial activity. Macrophages expressed receptor proteins MRC1, LGALS1, LRPAP1 and DMBT1L, mitochondria-localized proteins and arginine metabolism proteins. Following infection with *S.* Enteritidis, macrophages increased the expression of lysosomal and phagosomal proteins (CTSB, CTSC, RAB7A, CATHL1, RSFR, GAL1, SOD1).

Chicken macrophages differed from heterophils in 3 specific features. First, macrophages specifically expressed receptors such as MRC1L, LRP1, LGALS1, LRPAP1 and DMBT1L. Second, macrophages exhibited higher mitochondrial activity including fatty acid degradation, TCA cycle and oxidative phosphorylation. And third, macrophages specifically expressed enzymes involved in arginine and proline metabolism (Figure 1). Receptors specifically expressed by macrophages indicate their potential to sense signals from the external environment which allows them to modulate immune response [6, 7] including their own polarization [26, 27]. The dependency of macrophages on oxidative phosphorylation and mitochondria functions was already described for human macrophages and neutrophils [28]. Macrophages were also enriched in arginine and proline metabolism since one of their bactericidal activities is the production of NO radicals by iNOS and arginine [29]. Following infection with *S.* Enteritidis, macrophages increased the expression of lysosomal and phagosomal proteins what could be associated not only with *S.* Enteritidis inactivation but also with macrophage ability of antigen presentation.

Heterophils specifically expressed granular proteins MPO, LYZ, LYG2, RSFR, LECT2, CATHL1, CATHL2, CTSL1, CTSG, OTFB, SERPINB1 and MMP9, and endoplasmic reticulum proteins SSR1, PDIA4, PDIA6, PPIB, BiP, HSP90B1 and CANX. The latter group of proteins is activated when lumenal conditions in endoplasmic reticulum are altered or chaperone capacity is overwhelmed by unfolded or misfolded proteins [30]. Induction of an unfolded protein response leads to neutrophil degranulation in mice [31] and based on our results, a similar response can be predicted also in chicken heterophils.

Granular proteins decreased in heterophils in response to infection. Since transcription of genes encoding these proteins did not change and the number of ribosomal proteins increased, these genes must have remained continuously transcribed and translated even after initial degranulation [24, 32–35]. However, not all proteins that decreased in heterophils following *S.* Enteritidis infection were assigned to pathogen inactivation. Matrix metalloproteinase MMP9 is used for degradation of the extracellular matrix to enable leukocyte infiltration to the site of inflammation [36], and ALB and FN1, are found at the surface of granulocytes and inhibit their migration [37, 38]. The decrease of ALB and FN1 together with the degradation of extracellular matrix by MMP9 leads to heterophil translocation from the blood circulation to the site of inflammation.

**Figure 2:**
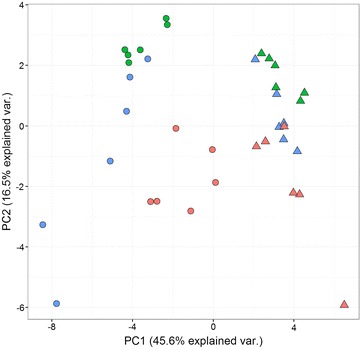
**PCA cluster analysis of chicken heterophils and macrophages using expression data from qPCR.** Each spot represents heterophils (circles) or macrophages (triangles) isolated from non-infected (green color), infected (red color), and vaccinated and infected chickens (blue color), 6 chickens per group. Heterophils from vaccinated chickens responded to infection more than macrophages from the same chicken. Transcription of heterophils and macrophages from naive but infected chickens approached the same profile.

Comparing expression at the protein and RNA levels provided several unexpected results. Changes in expression at the RNA level in response to infection were more pronounced in heterophils than in macrophages. We can exclude any technical issues in macrophage gene expression analysis since there were at least 3 genes inducible at the RNA level also in macrophages (AVD, MRP126 and F13A). Unlike macrophages, there were also greater differences in the expression profiles of heterophils obtained from vaccinated chickens in comparison to those obtained from naive but infected animals and an increase in CATHL2 and LECT2 in the heterophils from the vaccinated chickens following *S.* Enteritidis challenge appeared as a specific positive marker of vaccination. Despite this, expression in heterophils and macrophages in naive but infected chickens tended to approach a similar expression profile (Figure 2).

In this study we characterized protein expression in chicken heterophils and macrophages in response to intravenous infection with *S.* Enteritidis. Heterophils decreased ALB and FN1, and released MMP9 to enable their translocation to the site of infection. Secondly the endoplasmic reticulum proteins increased in heterophils which resulted in the release of granular proteins. On the other hand, macrophages were less responsive to acute infection and an increase in proteins like CATHL1, CATHL2, RSFR, LECT2 and GAL1 in the absence of any change in their expression at RNA level could even be explained by capturing these proteins from the external environment into which these could have been released by heterophils.

## Additional files

**Additional file 1.** **List of primers used in quantitative RT PCR in the study.**

**Additional file 2.** **Identification of heterophil and macrophage specific proteins using label-free LC MS/MS and PSM quantification.**

**Additional file 3.** **Heterophil proteins responding to in vivo infection with** ***S.*** **Enteritidis.**

**Additional file 4.** **All heterophil proteins quantified in this study.**

**Additional file 5.** **Macrophage proteins responding to in vivo infection with** ***S.*** **Enteritidis.**

**Additional file 6.** **All macrophage proteins quantified in this study.**

**Additional file 7.** **Expression of selected genes at RNA level determined by quantitative RT PCR.**

## References

[1] Silva MT (2010). When two is better than one: macrophages and neutrophils work in concert in innate immunity as complementary and cooperative partners of a myeloid phagocyte system. J Leukoc Biol.

[2] Genovese KJ, He H, Swaggerty CL, Kogut MH (2013). The avian heterophil. Dev Comp Immunol.

[3] Kogut MH, McGruder ED, Hargis BM, Corrier DE, DeLoach JR (1995). In vivo activation of heterophil function in chickens following injection with *Salmonella* Enteritidis-immune lymphokines. J Leukoc Biol.

[4] Kogut MH, Tellez G, Hargis BM, Corrier DE, DeLoach JR (1993). The effect of 5-fluorouracil treatment of chicks: a cell depletion model for the study of avian polymorphonuclear leukocytes and natural host defenses. Poult Sci.

[5] Barrow PA (2007). *Salmonella* infections: immune and non-immune protection with vaccines. Avian Pathol.

[6] Matulova M, Rajova J, Vlasatikova L, Volf J, Stepanova H, Havlickova H, Sisak F, Rychlik I (2012). Characterization of chicken spleen transcriptome after infection with *Salmonella enterica serovar* Enteritidis. PLoS One.

[7] Matulova M, Stepanova H, Sisak F, Havlickova H, Faldynova M, Kyrova K, Volf J, Rychlik I (2012). Cytokine signaling in splenic leukocytes from vaccinated and non-vaccinated chickens after intravenous infection with *Salmonella* Enteritidis. PLoS One.

[8] Qureshi MA (2003). Avian macrophage and immune response: an overview. Poult Sci.

[9] Swaggerty CL, Pevzner IY, Kaiser P, Kogut MH (2008). Profiling pro-inflammatory cytokine and chemokine mRNA expression levels as a novel method for selection of increased innate immune responsiveness. Vet Immunol Immunopathol.

[10] Singh R, Jain P, Pandey NK, Saxena VK, Saxena M, Singh KB, Ahmed KA, Singh RP (2012). Cytokines expression and nitric oxide production under induced infection to *Salmonella Typhimurium* in chicken lines divergently selected for cutaneous hypersensitivity. Asian-Australas J Anim Sci.

[11] Rychlik I, Karasova D, Sebkova A, Volf J, Sisak F, Havlickova H, Kummer V, Imre A, Szmolka A, Nagy B (2009). Virulence potential of five major pathogenicity islands (SPI-1 to SPI-5) of *Salmonella enterica serovar* Enteritidis for chickens. BMC Microbiol.

[12] Bustin SA, Benes V, Garson JA, Hellemans J, Huggett J, Kubista M, Mueller R, Nolan T, Pfaffl MW, Shipley GL, Vandesompele J, Wittwer CT (2009). The MIQE guidelines: minimum information for publication of quantitative real-time PCR experiments. Clin Chem.

[13] De Boever S, Vangestel C, De Backer P, Croubels S, Sys SU (2008). Identification and validation of housekeeping genes as internal control for gene expression in an intravenous LPS inflammation model in chickens. Vet Immunol Immunopathol.

[14] Li YP, Bang DD, Handberg KJ, Jorgensen PH, Zhang MF (2005). Evaluation of the suitability of six host genes as internal control in real-time RT-PCR assays in chicken embryo cell cultures infected with infectious bursal disease virus. Vet Microbiol.

[15] Wiśniewski JR, Zougman A, Nagaraj N, Mann M (2009). Universal sample preparation method for proteome analysis. Nat Methods.

[16] Boersema PJ, Raijmakers R, Lemeer S, Mohammed S, Heck AJ (2009). Multiplex peptide stable isotope dimethyl labeling for quantitative proteomics. Nat Protoc.

[17] Lundgren DH, Hwang SI, Wu L, Han DK (2010). Role of spectral counting in quantitative proteomics. Expert Rev Proteomics.

[18] Old WM, Meyer-Arendt K, Aveline-Wolf L, Pierce KG, Mendoza A, Sevinsky JR, Resing KA, Ahn NG (2005). Comparison of label-free methods for quantifying human proteins by shotgun proteomics. Mol Cell Proteomics.

[19] Wong JW, Sullivan MJ, Cagney G (2008). Computational methods for the comparative quantification of proteins in label-free LCn-MS experiments. Brief Bioinform.

[20] Adeva-Andany MM, González-Lucán M, Donapetry-García C, Fernández-Fernández C, Ameneiros-Rodríguez E (2016). Glycogen metabolism in humans. BBA Clin.

[21] Staines K, Hunt LG, Young JR, Butter C (2014). Evolution of an expanded mannose receptor gene family. PLoS One.

[22] Li YZ, Cheng CS, Chen CJ, Li ZL, Lin YT, Chen SE, Huang SY (2014). Functional annotation of proteomic data from chicken heterophils and macrophages induced by carbon nanotube exposure. Int J Mol Sci.

[23] Ye H, Xu FZ, Yu WY (2009). The intracellular localization and oligomerization of chicken invariant chain with major histocompatibility complex class II subunits. Poult Sci.

[24] van Dijk A, Molhoek EM, Veldhuizen EJ, Bokhoven JL, Wagendorp E, Bikker F, Haagsman HP (2009). Identification of chicken cathelicidin-2 core elements involved in antibacterial and immunomodulatory activities. Mol Immunol.

[25] van Dijk A, Tersteeg-Zijderveld MH, Tjeerdsma-van Bokhoven JL, Jansman AJ, Veldhuizen EJ, Haagsman HP (2009). Chicken heterophils are recruited to the site of *Salmonella* infection and release antibacterial mature Cathelicidin-2 upon stimulation with LPS. Mol Immunol.

[26] Novak R, Dabelic S, Dumic J (2012). Galectin-1 and galectin-3 expression profiles in classically and alternatively activated human macrophages. Biochim Biophys Acta.

[27] May P, Bock HH, Nofer JR (2013). Low density receptor-related protein 1 (LRP1) promotes anti-inflammatory phenotype in murine macrophages. Cell Tissue Res.

[28] Kramer PA, Ravi S, Chacko B, Johnson MS, Darley-Usmar VM (2014). A review of the mitochondrial and glycolytic metabolism in human platelets and leukocytes: implications for their use as bioenergetic biomarkers. Redox Biol.

[29] Hussain I, Qureshi MA (1997). Nitric oxide synthase activity and mRNA expression in chicken macrophages. Poult Sci.

[30] Lai E, Teodoro T, Volchuk A (2007). Endoplasmic reticulum stress: signaling the unfolded protein response. Physiology (Bethesda).

[31] Hu R, Chen ZF, Yan J, Li QF, Huang Y, Xu H, Zhang XP, Jiang H (2015). Endoplasmic reticulum stress of neutrophils is required for ischemia/reperfusion-induced acute lung injury. J Immunol.

[32] Rosenberg HF (2008). RNase A ribonucleases and host defense: an evolving story. J Leukoc Biol.

[33] Veldhuizen EJ, Brouwer EC, Schneider VA, Fluit AC (2013). Chicken cathelicidins display antimicrobial activity against multiresistant bacteria without inducing strong resistance. PLoS One.

[34] Johnson DA, Barrett AJ, Mason RW (1986). Cathepsin L inactivates alpha 1-proteinase inhibitor by cleavage in the reactive site region. J Biol Chem.

[35] Baumann M, Pham CT, Benarafa C (2013). SerpinB1 is critical for neutrophil survival through cell-autonomous inhibition of cathepsin G. Blood.

[36] Bradley LM, Douglass MF, Chatterjee D, Akira S, Baaten BJ (2012). Matrix metalloprotease 9 mediates neutrophil migration into the airways in response to influenza virus-induced toll-like receptor signaling. PLoS Pathog.

[37] Nathan C, Xie QW, Halbwachs-Mecarelli L, Jin WW (1993). Albumin inhibits neutrophil spreading and hydrogen peroxide release by blocking the shedding of CD43 (sialophorin, leukosialin). J Cell Biol.

[38] Everitt EA, Malik AB, Hendey B (1996). Fibronectin enhances the migration rate of human neutrophils in vitro. J Leukoc Biol.

